# Educational and health outcomes of children and adolescents receiving antiepileptic medication: Scotland-wide record linkage study of 766 244 schoolchildren

**DOI:** 10.1186/s12889-019-6888-9

**Published:** 2019-05-17

**Authors:** Michael Fleming, Catherine A. Fitton, Markus F. C. Steiner, James S. McLay, David Clark, Albert King, Daniel F. Mackay, Jill P. Pell

**Affiliations:** 1 Institute of Health and Wellbeing, University of Glasgow, 1 Lilybank Gardens, Glasgow, G12 8RZ UK; 2 Department of Child Health, University of Aberdeen, Aberdeen, AB25 2ZG UK; 3 Information Services Division, Edinburgh, EH12 9EB UK; 4 ScotXed, Scottish Government, Edinburgh, EH6 6QQ UK

**Keywords:** Epilepsy, Educational outcomes, Health, Population cohort, Record linkage, Prescribing

## Abstract

**Background:**

Childhood epilepsy can adversely affect education and employment in addition to health. Previous studies are small or highly selective producing conflicting results. This retrospective cohort study aims to compare educational and health outcomes of children receiving antiepileptic medication versus peers.

**Methods:**

Record linkage of Scotland-wide databases covering dispensed prescriptions, acute and psychiatric hospitalisations, maternity records, deaths, annual pupil census, school absences/exclusions, special educational needs, school examinations, and (un)employment provided data on 766,244 children attending Scottish schools between 2009 and 2013. Outcomes were adjusted for sociodemographic and maternity confounders and comorbid conditions.

**Results:**

Compared with peers, children on antiepileptic medication were more likely to experience school absence (Incidence Rate Ratio [IRR] 1.43, 95% CI: 1.38, 1.48), special educational needs (Odds ratio [OR] 9.60, 95% CI: 9.02, 10.23), achieve the lowest level of attainment (OR 3.43, 95% CI: 2.74, 4.29) be unemployed (OR 1.82, 95% CI: 1.60, 2.07), be admitted to hospital (Hazard Ratio [HR] 3.56, 95% CI: 3.42, 3.70), and die (HR 22.02, 95% CI: 17.00, 28.53). Absenteeism partly explained poorer attainment and higher unemployment. Girls and younger children on antiepileptic medication had higher risk of poor outcomes.

**Conclusions:**

Children on antiepileptic medication fare worse than peers across educational and health outcomes. In order to reduce school absenteeism and mitigate its effects, children with epilepsy should receive integrated care from a multidisciplinary team that spans education and healthcare.

## What is known on this subject?

Children who have epilepsy suffer increased morbidity and mortality and experience deficits in cognitive function. However, there is conflicting evidence as to whether their disadvantages extend to poorer school outcomes.

## What this study adds?

This population-wide record linkage study is the largest to date to investigate a wide range of both educational and health outcomes in an unselected cohort of children taking antiepileptic medication compared to their peers.

## Background

Epilepsy is a relatively common neurological condition for which medication is the main intervention to control seizure activity [1, 2]. In developed countries, such as Scotland, the childhood prevalence of epilepsy has been estimated at 3.2–9.3 per 1000 [3–6] and childhood incidence is highest in the first year of life. [3, 4] Childhood epilepsy is associated with increased risk of mortality [7–11] including sudden death in epilepsy (SUDEP) [12] and suicide [13], as well as morbidity and hospitalisation, especially due to injury. [14–17] Previous studies have demonstrated higher mortality rates among children with epilepsy compared to the general population, reporting increased risk of death between three and twenty-fold. [7–11]. However, to the best of our knowledge, previous studies have not directly compared children with epilepsy to a cohort of their unaffected peers with respect to all-cause mortality or all cause hospitalisation. Studies reporting hospitalisations in children with epilepsy compared to peers have focussed on injury admissions with associations evident in large studies [14] but inconclusive findings reported in smaller studies limited by sample size [15, 18]. Most commonly reported reasons for admission among children with epilepsy include fractures [14–16], head and dental injuries [15, 16], burns and scalds [14, 15], soft tissue injuries [16], poisoning [14], drowning and submersion [17] and self-harm [19].

In addition to poorer health outcomes, several studies have reported lower academic achievement among children with epilepsy compared to their unaffected peers [20–27], and children with other chronic conditions such as asthma. [28, 29] However, the majority of these studies have relied on battery tests, or parental or teacher reports of school grades [20–22, 24–29] and few have analysed official school recorded exam grades [23]. Furthermore, the evidence for worse academic outcomes is based on relatively few, small sample size studies [21, 22, 26–28, 30] many of which sampled children attending hospital clinics [21, 23, 25, 30]. Whether epilepsy does [28, 30, 31] or does not [21, 22] impact on intelligence is unclear. However, there is consistent evidence that epilepsy is associated with learning difficulties [21, 28, 32], reduced cognition [20, 33] and specific cognitive impairment relating to: attention [21, 32, 34, 35], memory [21, 31, 32, 36], dexterity [32, 34], psychomotor speed [31, 34], verbal function [37], executive function [26, 36], language [21, 32], perception [32], auditory processing [26], and response inhibition [34]. Children with epilepsy are also more likely to suffer comorbid conditions such as attention deficit hyperactivity disorder (ADHD) and conduct disorders [28, 31, 38, 39], anxiety [28], depression [40–42], low self-esteem [35, 43] and psychosocial dysfunction [44]. A limited number of small sample studies have reported higher rates of school dropout [28], increased use of special educational services [45] and increased school absenteeism [28, 30]; however, to our knowledge, none have reported school exclusion or unemployment. To the best of our knowledge, no population-wide studies have previously investigated associations between epilepsy and school outcomes and no such studies have been conducted in the UK. This retrospective cohort study fills several important gaps in the literature by using record linkage of eight Scotland-wide databases to study a range of education and health outcomes in an unselected countrywide cohort of schoolchildren taking antiepileptic medication compared to their peers.

## Methods

### Databases

Individual-level data from four Scotland-wide health databases and four Scotland-wide education databases were linked together using linkage methodology described previously. [46–48] Health data were provided by the Information Services Division (ISD) of the National Health Service (NHS) and education data were provided by the Scottish Exchange of Educational Data (ScotXed). The study cohort comprised singleton children born in Scotland who attended a Scottish local authority run school at any point between 2009 and 2013.

The prescribing information system (PIS) collects data on prescriptions dispensed by community pharmacies or primary care to Scottish residents. The Scottish Morbidity Record (SMR) 02 maternity database collects maternal and obstetric data pertaining to pregnant mothers’ and outcomes pertaining to their offspring. Acute and psychiatric hospital admissions are recorded through SMR01 and SMR04 records respectively, which include dates of admission and discharge and International Classification of Diseases (ICD) diagnostic codes. The National Records of Scotland collect date and cause of death from death certificates.

The pupil census, conducted every September by local authority run primary, secondary and special schools across Scotland, gathers pupil demographic information including whether the child has a special educational need and its type. The Scottish Qualifications Authority collects school exam results for these schoolchildren. Absences and exclusions are collected prospectively and appended to the school census at the end of each school year. The school leaver database collects information on whether pupils are in paid/voluntary employment, higher/further education, or training or are unemployed six months after leaving school.

### Inclusion criteria, definitions and outcomes

**Fig. 1:**
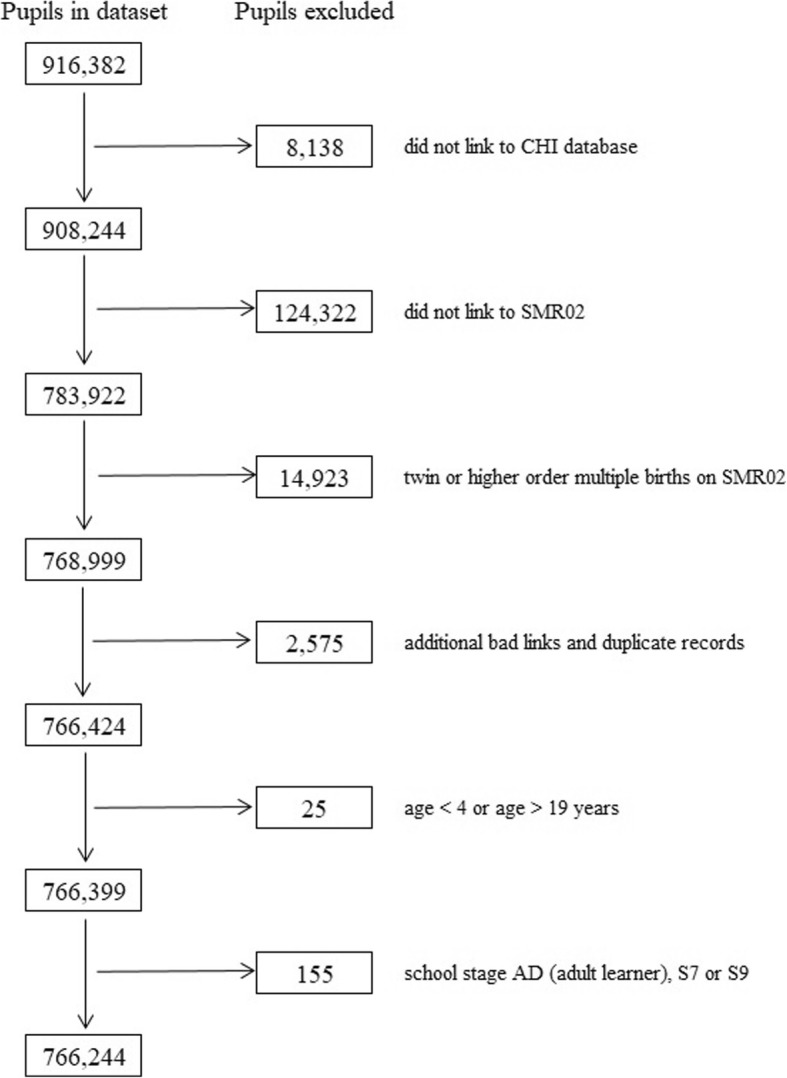
Number of Pupils Included and Excluded at Each Stage of Data Cleaning

Records pertaining to children aged < 4 years or > 19 years in the pupil census were excluded from the analyses. The final cohort comprised singleton births only because, for same sex multiple births, we could not be certain that the correct child had linked (Fig. 1). We used PIS data to identify children prescribed medication for epilepsy (any drug from BNF section 4.8), [47, 49] ADHD (methylphenidate hydrochloride, dexamphetamine sulphate, atomoxetine or lisdexamfetamine dimesylate), diabetes (insulin) or depression (tricyclic antidepressant, selective serotonin reuptake inhibitor, mirtazapine or venlafaxine) on at least one occasion over the school year and those prescribed medication for asthma (inhaled corticosteroid and beta agonist both dispensed twice or more over one year). This methodology has been described previously. [47, 48] The comparison group (peers) was all other children attending school over the study period (i.e. children not in receipt of antiepileptic medication).

Educational outcomes analysed were annual number of days absent, annual number of school exclusions, annual record and type of special educational need, leaving school before 16 years of age, final academic achievement, and subsequent unemployment. Data on absences and exclusions were only available for 2009, 2010 and 2012. The last three outcomes were only available for pupils who left school within the study period.

Special educational need is defined as inability to benefit from school education without additional help over and above that normally given to schoolchildren of the same age. The school census included special educational need ascribed to intellectual disabilities, learning difficulties, dyslexia, physical/motor/sensory impairment, language/speech disorder, autistic spectrum disorder, physical/mental health conditions and social/emotional/behavioural difficulties. Recording of more than one was allowed. An ordinal measure of academic achievement (low, basic, broad general and high attainment) over the last three years of school (S4-S6) was derived using the number of exam awards attained at each level of the Scottish Credit Qualifications Framework (SCQF). [50] Leaver destination was analysed as a binary variable (education/employment/training or unemployment). Health outcomes analysed were all-cause hospitalisation, hospitalisation for injury, poisoning or trauma (ICD-10 codes S00-T98), and all-cause mortality. We had follow up data on acute and psychiatric hospitalisations and deaths until September 2014 enabling a mean follow-up period of 4.3 years (range 1 to 5 years).

In addition to comorbid ADHD, depression, diabetes or asthma, identified from prescribing data, we adjusted our analyses for several other potential confounders. The pupil census recorded children’s sex, age and ethnicity. General population quintiles of area socioeconomic deprivation were derived for data zones of residence (median population 769) using the Scottish Index of Multiple Deprivation (SIMD) 2012, calculated from 38 indicators across 7 domains (health, housing, employment, income, crime and education, skills and training, and geographic access). Retrospective linkage to SMR02 enabled adjustment for maternal age at delivery, gestation at delivery, maternal smoking, mode of delivery, 5 min Apgar score, parity, and derived sex-, gestation-specific birthweight centiles.

### Statistical analyses

We used chi square tests for categorical data and chi square tests for trend for ordinal data to compare the characteristics of children on antiepileptic medication with their peers. Absences, exclusions and special educational need were recorded annually and analysed as yearly outcomes. We used generalised estimating equations (GEE) to account for correlations between observations repeated for the same pupil across different years. [51] We compared different correlation structures using the user-written quasi-likelihood under the independence model criterion (QIC) statistic. The lowest trace QIC indicated the most appropriate structure. [52] Number of days absent and number of exclusions were modelled using GEE analyses with a negative binomial distribution and log link function using number of possible attendances each year as an offset variable to adjust for individual exposure time. We modelled special educational need using GEE analyses with a binomial distribution and logit link. Age at leaving school and unemployment were analysed using binary logistic regression models and academic attainment was analysed using generalised ordinal logistic regression. Hospitalisation and mortality were modelled using Cox proportional hazard models where the assumption of proportionality held; otherwise, Poisson piecewise regression models were used. Tests for proportional hazards were conducted using the Stata *estat phtest* command. These longer-term end-outcomes were summarised and modelled on a pupil, rather than yearly, basis dependent on whether children had previously been prescribed epilepsy medication at any point within the study period. Therefore, longitudinal methods were not required. These methods have been described previously. [47, 48] We ran all models unadjusted, then adjusted for sociodemographic and maternity confounders and comorbid conditions: ADHD, depression, asthma and diabetes. We explored age, sex and deprivation as potential effect modifiers by firstly testing for statistical interactions and then undertaking sub-group analyses where interactions were significant. For academic attainment, we re-ran the multivariate models including absenteeism as a covariate to explore whether it was a mediator. For unemployment, we re-ran including both absenteeism and attainment as mediators. We also re-ran the attainment and unemployment models excluding children with special educational needs. All statistical analyses were undertaken using Stata MP version 14.1.

### Approvals

The authors applied for permission to access, link and analyse these data and undertook mandatory training in data protection, IT security and information governance. Therefore, the datasets generated and analysed during the study are not publicly available. The study was approved by the National Health Service Privacy Advisory Committee and covered by a data processing agreement between Glasgow University and ISD and a data sharing agreement between Glasgow University and ScotXed.

## Results

Between 2009 and 2013, 766,244 singleton children born in Scotland attended Scottish schools. Antiepileptic medication was used by 5314 (0.69%); more commonly by girls (0.72%) than boys (0.67%). Children on antiepileptic medication were more likely to live in deprived areas, and have mothers who were younger, smoked during pregnancy, and experienced pregnancy complications (Table 1). Compared with their peers, they were also more likely to be on medication for depression (8.54% versus 0.64%, *P* < 0.001) and ADHD (3.33% versus 0.95%, *P* < 0.001).

**Table 1:** Characteristics of Schoolchildren by Presence or Absence of Treated Epilepsy

	No epilepsy		Epilepsy		*P* value
	*N* = 760,930		*N* = 5314		
	N	%	N	%	
Sociodemographic factors					
Sex					
Male	387,672	50.9	2618	49.3	0.015
Female	373,258	49.1	2696	50.7	
Missing	0		0		
Deprivation quintile					
1 (most deprived)	172,422	22.7	1372	25.8	< 0.001
2	152,434	20.0	1135	21.4	
3	146,897	19.3	1027	19.3	
4	148,538	19.5	985	18.5	
5 (least deprived)	140,049	18.4	791	14.9	
Missing	590		4		
Ethnic group					
White	723,055	96.2	5061	96.2	< 0.001
Asian	17,641	2.3	136	2.6	
Black	1956	0.3	9	0.2	
Mixed	6684	0.9	44	0.8	
Other	2066	0.3	10	0.2	
Missing	9528		54		
Medication for comorbid conditions					
Diabetes	3278	0.4	52	1.0	< 0.001
Asthma	45,397	6.0	503	9.5	< 0.001
ADHD	7236	1.0	177	3.3	< 0.001
Depression	4888	0.6	454	8.5	< 0.001
Maternity factors					
Maternal age (years)					
≤24	208,289	27.4	1589	29.9	< 0.001
25–29	222,902	29.3	1638	30.8	
30–34	215,540	28.3	1395	26.3	
≥35	114,187	15.0	692	13.0	
Missing	12		0		
Maternal smoking					
No	487,860	72.4	3254	69.9	< 0.001
Yes	186,387	27.6	1402	30.1	
Missing	86,683			658	
Parity					
0	343,228	45.3	2437	46.0	0.400
1	262,333	34.7	1809	34.2	
> 1	151,521	20.0	1049	19.8	
Missing	3848		19		
Mode of delivery					
SVD	512,788	67.4	3431	64.6	< 0.001
Assisted vaginal	91,039	12.0	618	11.6	
Breech vaginal	2211	0.3	22	0.4	
Elective CS	57,907	7.6	407	7.7	
Emergency CS	96,822	12.7	834	15.7	
Other	161	0.0	2	0.0	
Missing	2		0		
Gestation (weeks)					
< 24	27	0.0	2	0.0	< 0.001
24–27	1087	0.1	38	0.7	
28–32	6935	0.9	123	2.3	
33–36	35,255	4.6	347	6.5	
37	37,279	4.9	340	6.4	
38	95,243	12.5	749	14.1	
39	157,648	20.7	1092	20.6	
40	228,958	30.1	1472	27.7	
41	170,243	22.4	950	17.9	
42	26,934	3.5	190	3.6	
43	624	0.1	6	0.1	
> 43	140	0.0	0	0.0	
Missing	557		5		
Sex-gestation-specific birthweight centile					
1–3	31,173	4.1	313	5.9	< 0.001
4–10	68,129	9.0	517	9.7	
11–20	90,686	11.9	662	12.5	
21–80	447,092	58.8	3029	57.1	
81–90	64,956	8.5	407	7.7	
91–97	40,956	5.4	265	5.0	
98–100	16,969	2.2	110	2.1	
Missing	969		11		
5-min Apgar					
1–3	3619	0.5	90	1.7	< 0.001
4–6	7181	1.0	121	2.3	
7–10	742,359	98.6	5053	96.0	
Missing	7771		50		

*ADHD* attention deficit hyperactivity disorder, *N* number, *SVD* spontaneous vaginal delivery, *CS* Caesarean section

The subgroup analyses of absence and exclusion included 702,210 children. Children on antiepileptic medication had more days absent especially among younger children and girls. (Table 2). The magnitude of the relative association decreased with increasing deprivation (all interactions, *P* < 0.001): However, this was attributable to greater baseline absenteeism among the most deprived children not on antiepileptic medication compared to the least deprived (median 11.0 versus 5.5 days). Among children on antiepileptic medication, absenteeism was also higher for children in the most compared to the least deprived quintile (median 13.5 versus 8.5 days). Epilepsy was not significantly associated with number of exclusions from school on univariate analysis (IRR 1.19, 95% CI: 0.98, 1.44) or after adjusting for confounders (IRR 0.89, 95% CI: 0.73, 1.09).

**Table 2:** Association Between Treated Epilepsy and School Absence: Overall and by Sex, Age and Area Deprivation

	Univariate		Multivariable^a^		Multivariable^b^	
	IRR	95% CI	IRR	95% CI	IRR	95% CI
Overall	1.62	1.56,1.67	1.50	1.45,1.55	1.43	1.38,1.48
Boys	1.52	1.45,1.59	1.39	1.33,1.46	1.34	1.28,1.41
Girls	1.72	1.64,1.81	1.61	1.53,1.69	1.51	1.44,1.59
< 11 years	1.70	1.61,1.79	1.66	1.56,1.76	1.63	1.53,1.73
11-14 years	1.49	1.40,1.58	1.51	1.42,1.60	1.44	1.37,1.53
> 14 years	1.36	1.29,1.43	1.37	1.30,1.45	1.25	1.19,1.32
1 (most deprived)	1.29	1.22,1.37	1.23	1.16,1.30	1.18	1.11,1.25
2	1.51	1.40,1.62	1.44	1.35,1.54	1.37	1.28,1.47
3	1.77	1.63,1.91	1.61	1.48,1.75	1.54	1.41,1.67
4	1.83	1.68,1.99	1.63	1.50,1.77	1.54	1.41,1.67
5 (least deprived)	2.01	1.82,2.21	1.81	1.64,1.99	1.70	1.54,1.87

^a^adjusted for age, sex, deprivation quintile, ethnic group, maternal age, maternal smoking, parity, mode of delivery, gestation at delivery, sex- gestation-specific birthweight centile and 5-min Apgar score

^b^also adjusted for comorbid conditions (diabetes, asthma, attention deficit hyperactivity disorder and depression)

*IRR* Incidence Rate Ratio, *CI* confidence interval

All *p* < 0.001

Children on antiepileptic medication were more likely to have special educational needs on univariate analysis (OR 9.83, 95% CI: 9.29, 10.40) and following adjustment for sociodemographic and maternity factors (OR 10.11, 95% CI: 9.51, 10.75) and comorbid conditions (OR 9.60, 95% CI: 9.02, 10.23). The associations were stronger in girls (fully adjusted OR 11.06, 95% CI: 10.13, 12.07) than boys (fully adjusted OR 8.38, 95% CI: 7.68, 9.15) and stronger in younger children: < 11 years of age (fully adjusted OR 13.15, 95% CI: 11.89, 14.53) compared with > 14 years (fully adjusted OR 7.90, 95% CI: 7.26, 8.59). The association was stronger in the least deprived quintile (fully adjusted OR 14.58, 95% CI: 12.40, 17.13) than the most (fully adjusted OR 7.57, 95% CI: 6.73, 8.51); explained by special educational need among children not on anti-epileptic mediation already being more common in the most deprived quintile than the least (all interactions, *P* < 0.001). Among children not taking antiepileptic medication, 19.4% of the most deprived quintile had a special education need compared with 10.2% of the least deprived. Among children taking antiepileptic medication, special education need was still more common in the most deprived quintile than the least deprived: 60.3% versus 49.9% respectively. Taking antiepileptic medication was most strongly associated with special education need due to: a physical health condition (fully adjusted OR 59.78, 95% CI: 54.59, 65.46), physical motor disability (fully adjusted OR 55.79, 95% CI: 50.77, 61.30); sensory impairment (fully adjusted OR 29.77, 95% CI: 26.38, 33.59); learning disability (fully adjusted OR 27.11, 95% CI: 24.95, 29.46); and communication problems (fully adjusted OR 24.25, 95% CI: 21.91, 26.83).

The subgroup analyses of academic attainment included 139,205 children. Children on antiepileptic medication were significantly more likely to attain the lowest level of academic achievement univariately (OR 2.58, 95% CI: 2.12, 3.14) and after adjustment for sociodemographic and maternity factors (OR 4.07, 95% CI: 3.26, 5.08) and comorbid conditions (OR 3.43, 95% CI: 2.74, 4.29). Adjustment for absenteeism, attenuated the association but it remained statistically significant (fully adjusted OR 2.73, 95% CI: 2.12, 3.53). The relative impact was less in the most deprived children (fully adjusted OR 1.89, 95% CI: 1.43, 2.50) than the least deprived (fully adjusted OR 6.57, 95% CI: 3.30, 13.07) (interaction, *P* = 0.009). However, this was again due to higher absolute risk among unaffected children in deprived areas. Among children not on antiepileptic medication, 9.51% in the most deprived quintile achieved the lowest level of attainment compared with 1.03% in the least deprived. Among children on antiepileptic medication, low academic attainment was still more common among deprived children: 14.02% versus 6.47% respectively. When children with special educational need were excluded, children on antiepileptic medication were still more likely to attain the lowest level of academic achievement (fully adjusted OR 2.74, 95% CI: 1.96, 3.84).

Children on antiepileptic medication were less likely, than their peers, to quit school prior to 16 years of age (26.33% versus 28.83%); fully adjusted OR 0.83, 95% CI: 0.74, 0.93). However, they were more likely to be unemployed six months after leaving school univariately (OR 1.85, 95% CI: 1.64, 2.09) and after adjustment for sociodemographic and maternity factors (OR 1.99, 95% CI: 1.75, 2.25) and comorbid conditions (OR 1.82, 95% CI: 1.60, 2.07). The association was attenuated, but still present, after excluding children with special educational need (fully adjusted OR 1.33, 95% CI: 1.10, 1.61). When attainment was added to the models, it attenuated the associations and they were no longer statistically significant either including (fully adjusted 0.92, 95% CI: 0.73, 1.15, *P* = 0.448) or excluding (fully adjusted OR 1.07, 95% CI: 0.81, 1.41, *P* = 0.636) children with special educational need. The relative association was stronger in the least deprived quintile (fully adjusted OR 2.35, 95% CI: 1.63, 3.37) and was not statistically significant in the most deprived (fully adjusted OR 1.19, 95% CI: 0.93, 1.53, *P* = 0.171) (interaction, *P* < 0.001). Again, this reflected the underlying absolute risk. Among children not taking antiepileptic medication, 16.91% of the most deprived quintile were unemployed following school, compared with only 4.94% of the least deprived. Among children on antiepileptic medication, the corresponding figures were 18.54% and 12.17% respectively.

**Fig. 2:**
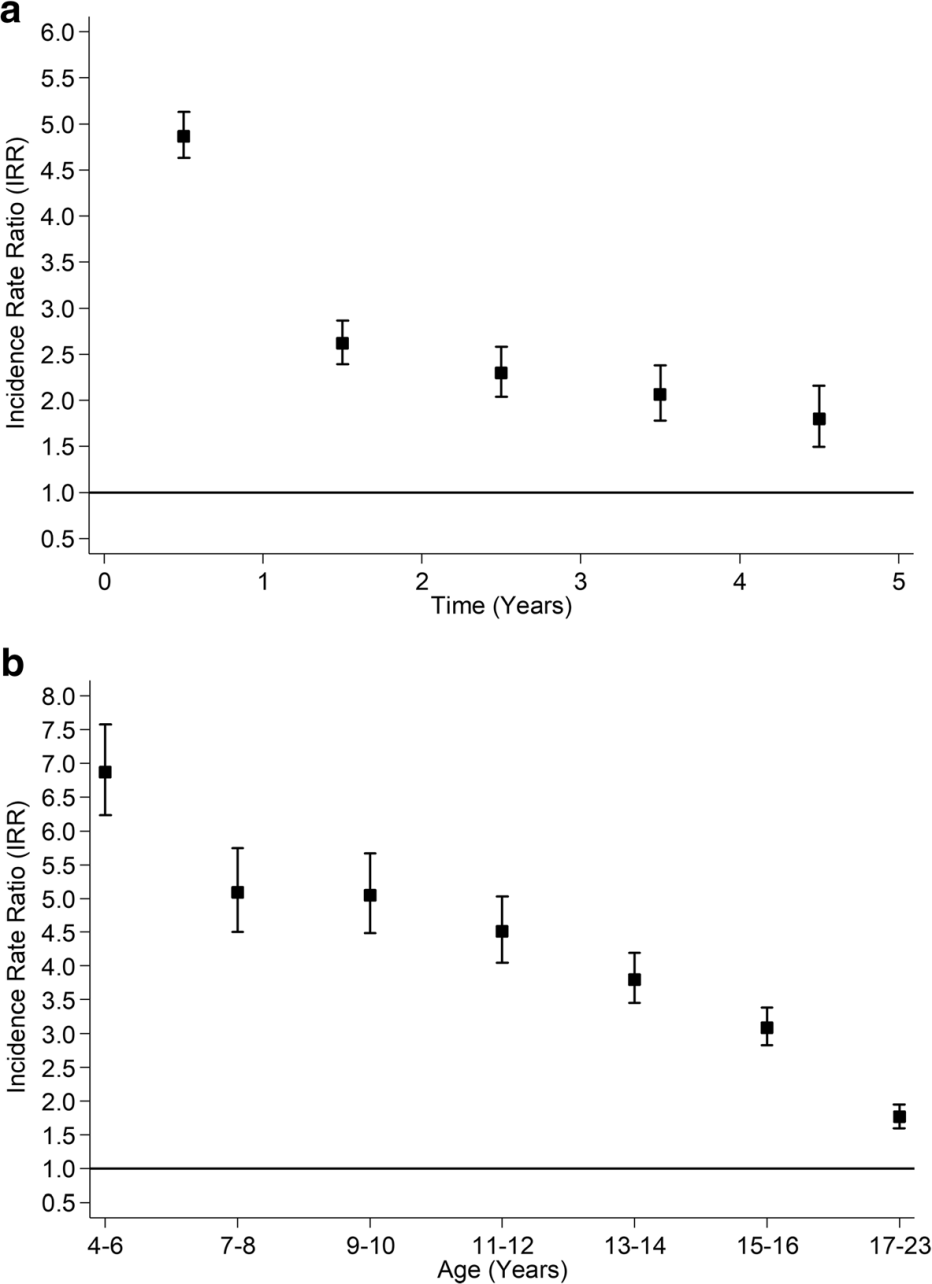
Association between Treatment with Antiepileptic Medication and all-cause Hospitalisation. **a** By Time from Commencement of Medication. **b** By Age at Admission. Adjusted for age, sex, deprivation quintile, ethnic group, maternal age, maternal smoking, parity, mode of delivery, gestation at delivery, sex- gestation-specific birthweight centile, 5-minute Apgar score and comorbid conditions (diabetes, asthma, attention deficit hyperactivity disorder and depression)

**Fig. 3:**
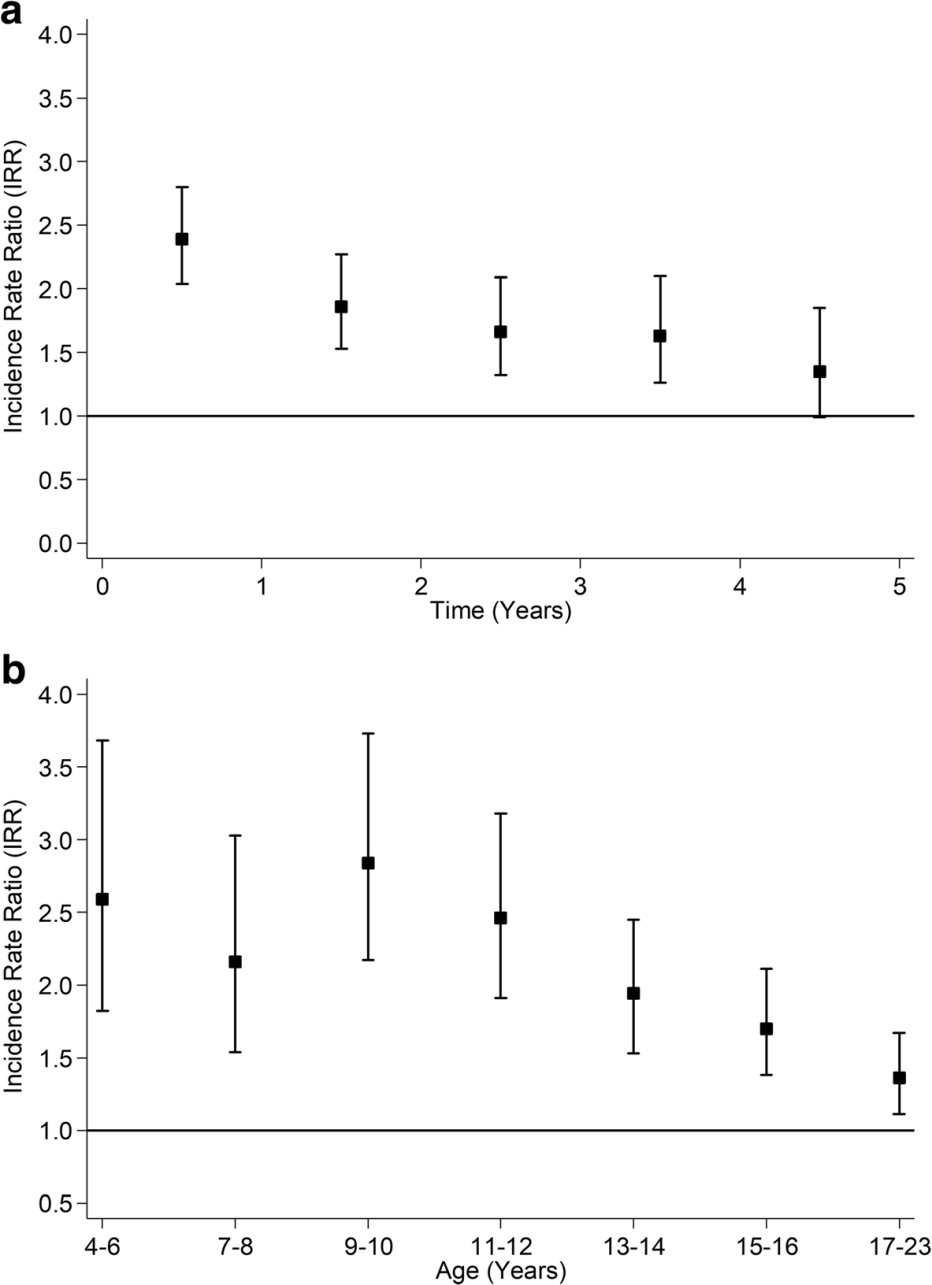
Association between Treatment with Antiepileptic Medication and Hospitalisation for Injury/Poisoning/ Trauma. **a** By Time from Commencement of Medication. **b** By Age at Admission. Adjusted for age, sex, deprivation quintile, ethnic group, maternal age, maternal smoking, parity, mode of delivery, gestation at delivery, sex- gestation-specific birthweight centile, 5-minute Apgar score and comorbid conditions (diabetes, asthma, attention deficit hyperactivity disorder and depression)

Over a mean of 4.3 years follow-up (range 1 to 5 years), 157,350 (20.5%) children were hospitalised at least once. Injury, poisoning or trauma accounted for 16.4% of hospitalisations in children on antiepileptic medication; 8.2% of children on antiepileptic medication had at least one hospital admission for injury, poisoning or trauma compared to 4.4% of their peers. In the Cox models, children on antiepileptic medication were more likely to be hospitalised for any cause (fully adjusted HR 3.56, 95% CI: 3.42, 3.70) and because of injury, poisoning or trauma (fully adjusted HR 1.88, 95% CI: 1.71, 2.07). However, the assumption of proportional hazards did not hold in either model (both *P* < 0.001). Therefore, Poisson piecewise regression models were run by period of follow-up (Figs. 2a & 3a) and by age of child at admission (Figs. 2b & 3b). Children on antiepileptic medication were at higher risk of hospitalisation throughout but the risk was highest in the first year after commencing medication (Fig. 2a) and at younger ages (Fig. 2b).

**Fig. 4:**
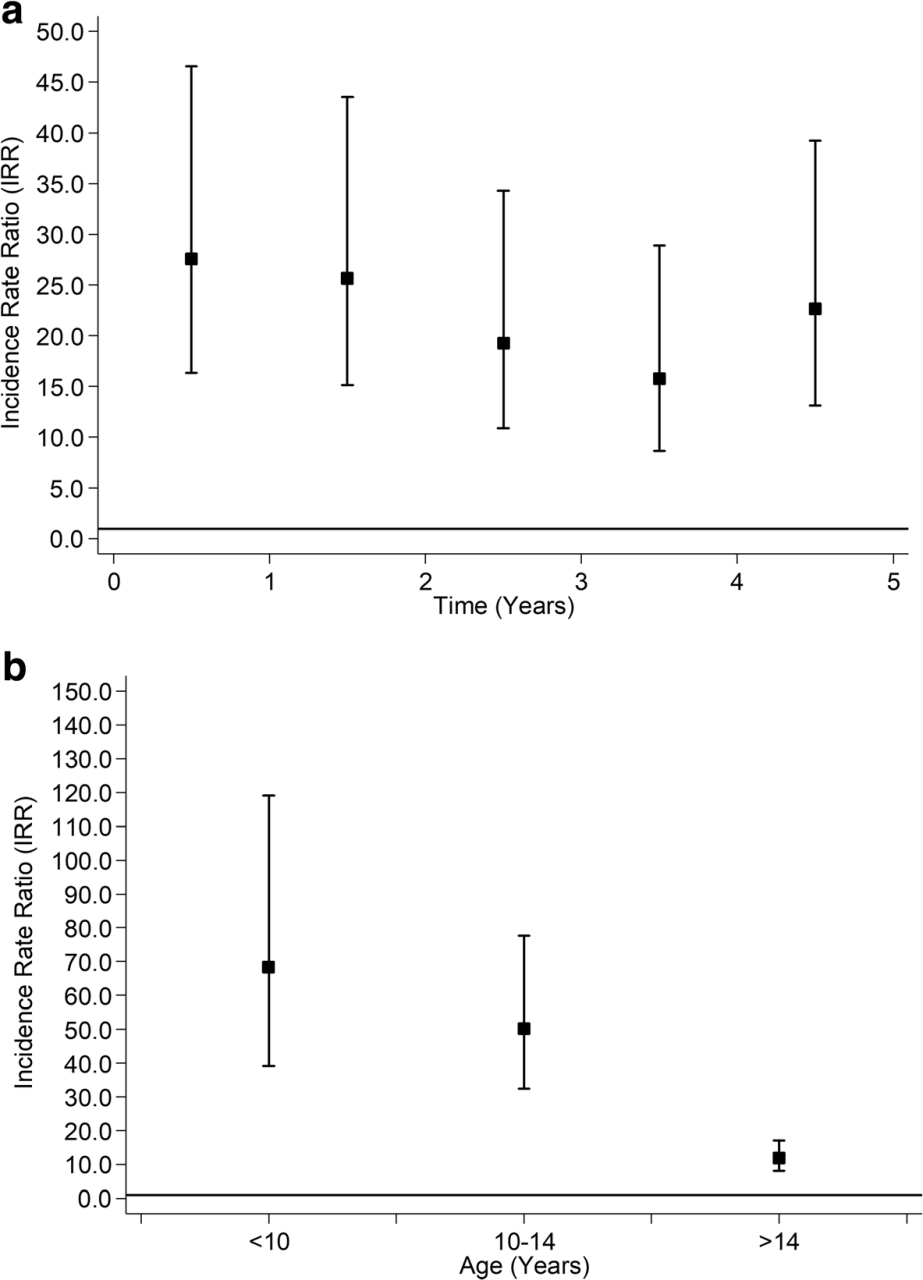
Association between Treatment with Antiepileptic Medication and all-cause Mortality. **a** By Time from Commencement of Medication. **b** By Age at Admission. Adjusted for age, sex, deprivation quintile, ethnic group, maternal age, maternal smoking, parity, mode of delivery, gestation at delivery, sex- gestation-specific birthweight centile, 5-minute Apgar score and comorbid conditions (diabetes, asthma, attention deficit hyperactivity disorder and depression)

Over follow-up, 491 children died; 81 deaths occurred among children treated for epilepsy and 410 among their peers. Among children treated for epilepsy 39.5% of deaths were attributed to diseases of the nervous system, 23.5% to congenital and chromosomal abnormalities, and 2.5% to injury. Among their peers, 44.4% of deaths were attributed to injury, 4.6% to diseases of the nervous system, and 4.6% to congenital and chromosomal abnormalities. The risk of death was higher among children on antiepileptic medication (fully adjusted HR 22.02, 95% CI: 17.00, 28.53). However, the assumption of proportional hazards again did not hold (*P* < 0.001) and, on Poisson piecewise regression analyses, the increased risk of death was highest below 14 years of age and within two years of commencing antiepileptic medication (Figs. 4a & 4b).

## Discussion

Children on antiepileptic medication fared worse than peers across a wide range of outcomes. In addition to increased hospitalisation and mortality they were at higher risk of: school absence, special educational need, poorer examination results and unemployment. Poorer attainment was partly explained by increased absenteeism and, in turn, explained higher rates of unemployment. Antiepileptic medication was more common among girls and had stronger associations with absenteeism and special educational need among girls. Younger children, and those who started medication more recently, also had higher risk.

Studies have consistently demonstrated higher mortality among children with epilepsy; ranging from 3 to 20-fold. [7–11] The most similar study to our own, using data from 430 general practices covering 5% of the UK population, reported an excess risk of similar magnitude to our study. [7] It is unclear whether higher mortality is restricted to those with severe underlying neurological conditions, [8] or not. [53] To the best of our knowledge, ours is the first study to compare all-cause hospitalisations among children taking antiepileptic medication with a cohort of their peers. Whereas previous studies have focussed specifically on injuries, [14, 15] we also reported all-cause hospitalisations. Our findings relating to injury were of comparable magnitude to those reported in a study using general practice data on 11,934 children prescribed antiepileptic medication and 46,598 matched peers [14]; however, we also demonstrated an even greater increased risk of all-cause hospitalisation.

A previous review identified 15 small-scale studies of academic achievement among epileptic children attending hospital and most used academic battery tests. [20] A few, small-scale studies, comprising between 73 and 116 children with epilepsy, have demonstrated poorer school performance. [23–25, 28] All but one [23] relied on parental or teacher reports of academic performance; one used sibling controls but did not match or adjust for gender [28] and two used neither matching or statistical adjustment to control for any potential confounders. [23, 24] Poorer educational attainment may be due to a number of mechanisms. Previous studies have produced conflicting results on whether epileptic children have lower, [28, 30, 31] or comparable, [21, 22] intelligence to their peers; perhaps reflecting heterogeneity in participant selection. Our finding of increased special educational need is consistent with previous reports of increased learning difficulties [21, 28, 32], reduced cognitive function [20, 33] and deficits in: attention [21, 32, 34, 35], memory [21, 31, 32, 36], language [21, 32], psychomotor speed [31, 34], dexterity [32, 34], perception [32], verbal function [37], auditory processing [26], response inhibition [34] and executive function. [26, 36] It has been suggested that poorer attainment may be confounded by, or mediated through, co-existence of ADHD and other conduct disorders [28, 31, 38, 39], depression, [40–42] anxiety, [28], psychosocial dysfunction [44] or low self-esteem. [35, 43] Our study confirmed that children on antiepileptic medication were more likely to be on medication for depression and ADHD. However, adjusting for these made little difference to the effect sizes and we demonstrated that attainment was lower even among children on antiepileptic medication who did not have a record of special educational need.

To the best of our knowledge, ours is the first study to demonstrate that children on antiepileptic medication were more likely to be unemployed six months after leaving school. Absenteeism appeared to partly mediate both poorer attainment and unemployment. Our finding of higher rates of absenteeism is consistent with some [28, 30] but not all [21] previous studies. Conversely, our finding of lower likelihood of leaving school before 16 years of age contrasts with previous evidence of higher rates of school dropout. [28]

Our observed associations with absenteeism, special educational need, hospitalisation and mortality were stronger in younger children. Epilepsy presenting at an early age is more likely to be associated with congenital neurological disability. [54] Also, younger children may have been diagnosed more recently and we demonstrated that risk of hospitalisation and death fell with time as children became stabilised on their medication. [55] The associations with absenteeism and special educational need were stronger in girls than boys. This finding requires corroboration and further exploration.

Ours was a large, non-selective study that covered the whole of Scotland. Ascertainment using school, rather than health, records ensured children with well controlled epilepsy were not excluded from the study. Use of antiepileptic medication is an indirect method of ascertaining epilepsy. Compared with self, parental or teacher report it is more objective and less prone to bias. Reliance on parental or teacher report may include children who had a single seizure, are not yet diagnosed with epilepsy, or do not have it. Since medication is the main intervention to control seizures incomplete ascertainment should be low. However, 30–43% of children may not comply with antiepileptic medication by discontinuing therapy within two years [56]. Previous studies using medication as a proxy measure of epilepsy have relied on issued prescriptions. [7, 14] Our data on encashed prescriptions are more likely to reflect actual usage.

The large study population was sufficiently powered to enable us to test for statistical interactions and conduct sub-group analyses, and we were able to analyse a wide range of educational and health outcomes in the same cohort. We could ensure that use of antiepileptic medication predated education and health outcomes and we adjusted for a wide range of potential confounders; however, residual confounding is possible in any observational study. Whilst our study only included children attending local authority maintained schools, only 5% of children in Scotland attend private schools. We could not link 12% of schoolchildren to their maternity records; however, this is consistent with the 11% of residents in Scotland aged 5–19 years who, according to the 2011 Scottish Census, were born out with Scotland. The prevalence of antiepileptic medication use among linked and unlinked pupils was 0.6% and 0.7% respectively, suggesting that bias was unlikely. Whilst our study used administrative databases established for other purposes, these datasets undergo strict quality assurance. We used probabilistic record linkage to match education and health records together and these methods have previously been validated and shown to be 99% accurate for singletons births. [46] A limitation of this study is that we identified cases of epilepsy based on administration of anti epileptic drugs, and it is not possible to deduce the type or severity of epilepsy from this information because clinicians tend to prescribe the most general anticonvulsant with the widest spectrum of activity. It is however likely that the educational and health outcomes associated with epilepsy may vary by type and severity of epilepsy and this merits further investigation in future studies where this information is available.

## Conclusions

Children on antiepileptic medication fared worse than peers across a wide range of outcomes. In addition to increased hospitalisation and mortality they were at higher risk of: school absence, special educational need, poorer examination results and unemployment. Poorer attainment was partly explained by increased absenteeism and, in turn, explained higher rates of unemployment. In order to reduce school absenteeism and mitigate its effects, children with epilepsy should receive integrated care from a multidisciplinary team covering physicians, teachers, parents, educational psychologists, and social services as appropriate. [57, 58] Their management should extend beyond healthcare to a programme of school-based interventions such as prevention of triggers, a seizure plan, social and emotional support, adaptation of teaching methods, resources and exams, and inclusion of classmates in cooperative learning. [58]

## Abbreviations

ADHD: Attention Deficit Hyperactivity Disorder
CS: Caesarean Section
HR: Hazard Ratio
ICD: International Classification of Diseases
IRR: Incidence Rate Ratio
ISD: Information Services Division
NHS: National Health Service
OR: Odds Ratio
PIS: Prescribing Information System
ScotXed: Scottish Exchange of Educational Data
SIMD: Scottish Index of Multiple Deprivation
SVD: Spontaneous Vaginal Delivery
